# Polyene Macrolactams from Marine and Terrestrial Sources: Structure, Production Strategies, Biosynthesis and Bioactivities

**DOI:** 10.3390/md20060360

**Published:** 2022-05-27

**Authors:** Wei Zhao, Hong Jiang, Xiao-Wan Liu, Jian Zhou, Bin Wu

**Affiliations:** 1 Polytechnic Institute, Zhejiang University, Hangzhou 310015, China; 12034024@zju.edu.cn; 2 Fujian Provincial Key Laboratory of Screening for Novel Microbial Products, Fujian Institute of Microbiology, Fuzhou 350007, China; jianghong709@163.com (H.J.); zjian503@163.com (J.Z.); 3 The State Key Laboratory of Marine Pollution and Department of Biomedical Sciences, City University of Hong Kong, Hong Kong SAR 999077, China; xiaowliu5-c@my.cityu.edu.hk; 4 Ocean College, Zhejiang University, Zhoushan 321000, China

**Keywords:** polyene macrolactams (PMLs), structural features, acquisition strategies, biosynthetic pathways, mechanisms, challenges and opportunities

## Abstract

Over the past few decades (covering 1972 to 2022), astounding progress has been made in the elucidation of structures, bioactivities and biosynthesis of polyene macrolactams (PMLs), but they have only been partially summarized. PMLs possess a wide range of biological activities, particularly distinctive fungal inhibitory abilities, which render them a promising drug candidate. Moreover, the unique biosynthetic pathways including β-amino acid initiation and pericyclic reactions were presented in PMLs, leading to more attention from inside and outside the natural products community. According to current summation, in this review, the chem- and bio-diversity of PMLs from marine and terrestrial sources are considerably rich. A systematic, critical and comprehensive overview is in great need. This review described the PMLs’ general structural features, production strategies, biosynthetic pathways and the mechanisms of bioactivities. The challenges and opportunities for the research of PMLs are also discussed.

## 1. Introduction

Microorganisms have a remarkable capacity for producing a wide range of unique bioactive secondary metabolites. A substantial number of them have developed into a supply of lead medicines, diagnostic reagents and biological probes. Among diverse microorganisms, *Streptomyces* and *Micromonospora* in the Actinomycetes family showed great potential in the discovery of drug leads or drug candidates. For instance, *Streptomyces*, a Gram-positive bacteria, is a rich reservoir of natural products [1,2], including anthraquinone (e.g., adriamycin), lactones (e.g., rapamycin), flavonoids (e.g., O-methylated phenylpropanoids) [3] and so on. *Micromonospora*, a genus of *Micromonosporaceae*, witnessed the recent growth of new actinomycetes-derived compounds, such as macrolides, spirotetronates, ansa-macrolides, angucycline, non-ribosomal peptide synthase (NPRS), PKS-NRPS hybrids and terpenes [2]. Both the two genera of bacteria are the main producers of polyene macrolactams (PMLs), which is a relatively small but growing family. PMLs exhibited a large spectrum of bioactivities such as antimicrobial (bombyxamycin A-C (**11**,**12**,**42**) [4], BE-14106 (**22**) [5] and ML449 (**28**) [6], antifungal (auroramycin (**37**) [7]), antitumor (vicenistatin (**32**) [8] and leinamycin (**48**) [9]) and anti-parasitic activities (lobosamides (**13**–**15**) [10] and sagamilactam (**73**) [11]), modulation of Bcl-xl function (incednine (**35**) [12]) and anti-inflammatory properties (cyclamenol A (**1**) [13]) (Table 1). Some typical PMLs compounds such as hitachimycin (**49**), BE-14106 (**22**), sceliphrolactam (**17**), heronamides (**25**–**27**, **62**–**72**), cremimycin (**80**) and macrotermycins (**30**–**31**,**76**,**79**) produced by *Streptomyces* sp. had impressive antifungal properties.

With the development of resistance to azoles [14,15,16], amphotericin B [17] is often considered the last defensive line for life-threatening fungal infections but its application is limited because of its high cytotoxicity. In the clinic, most antifungal agents target ergosterol biosynthetic pathways with only a few showing no relation to ergosterol targets [18]. It is encouraging that some PMLs with antifungal abilities have a unique antifungal mechanism. Although Alvarez R. et al. reviewed bio and chemical synthetic pathways of PMLs, there are only a few comprehensive reviews on PMLs [19]. Here, we provided an updated review on the structural classification, microbial sources, production strategies, biosynthetic pathways and bioactivities of PMLs. The remaining challenges and future opportunities are also discussed.

## 2. PMLs Structure

**Figure 1:**
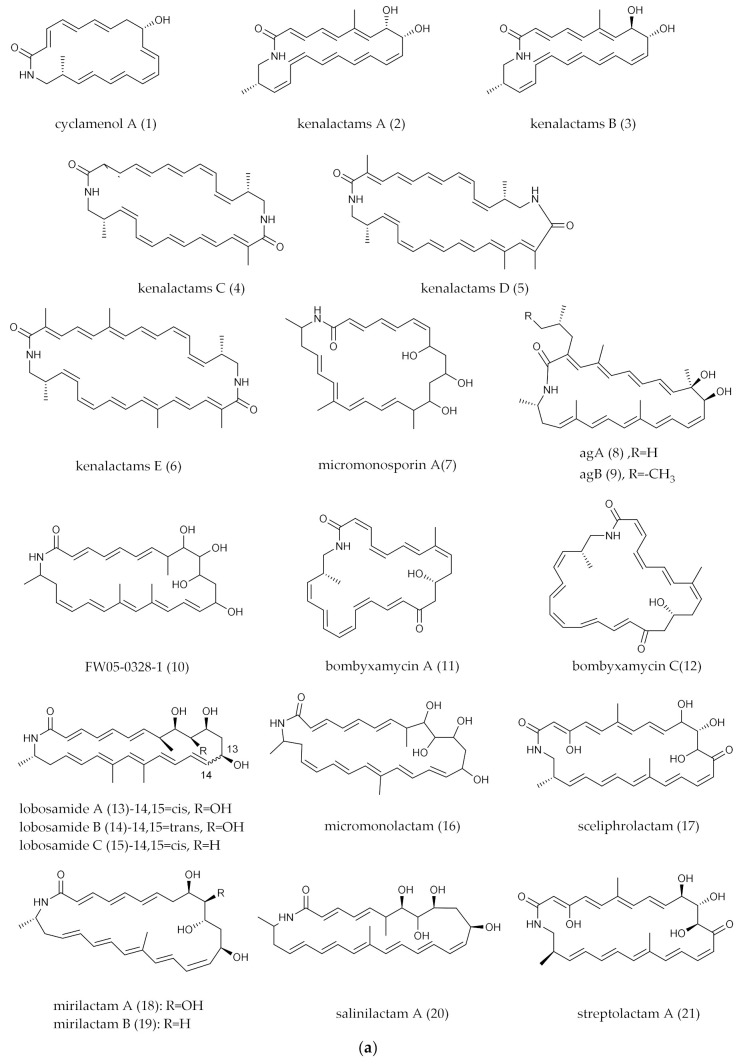

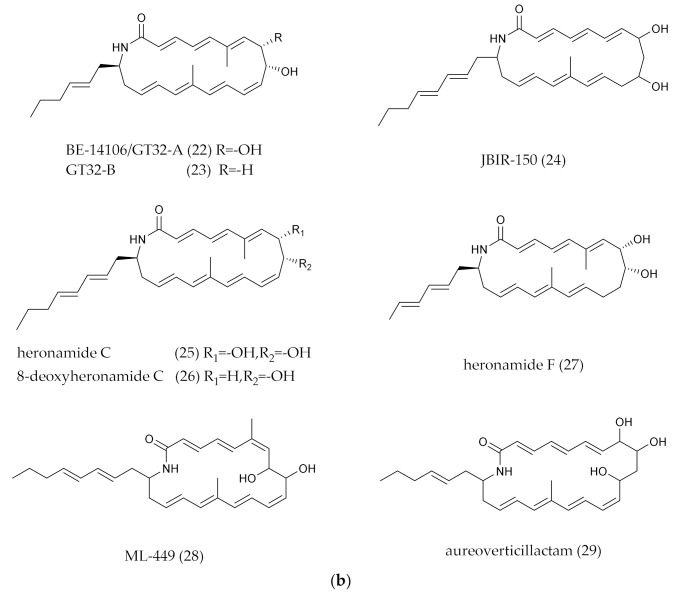

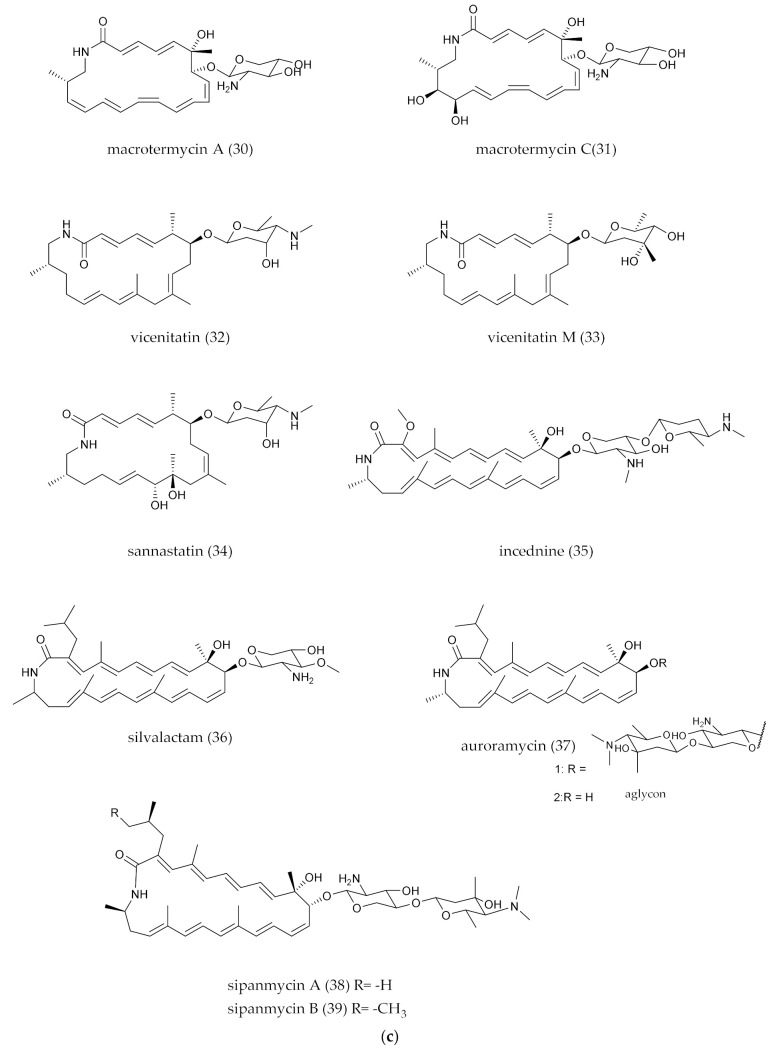

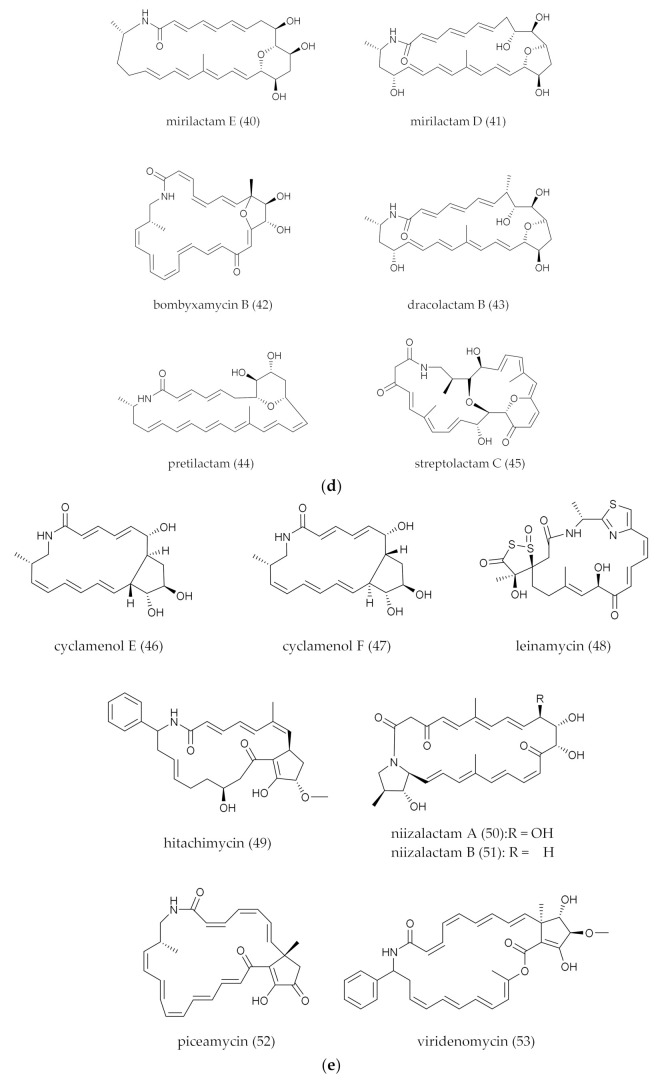
(**a**) Mono-ring PMLs (the substituents in the mono-ring of PMLs generally contain polyene and amide groups as well as polyhydroxyl and methyl groups). (**b**) Mono-ring PMLs with fatty acid chain. (**c**) Mono-ring PMLs with glycosylated groups. (**d**) Mono-ring PMLs with intramolecular epoxidation groups. (**e**) Mono-ring PMLs containing a five-membered ring.

**Figure 2:**
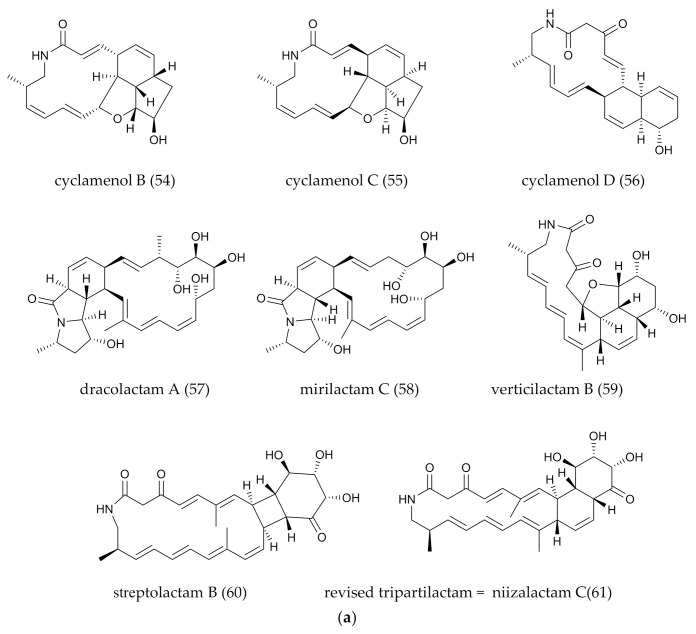

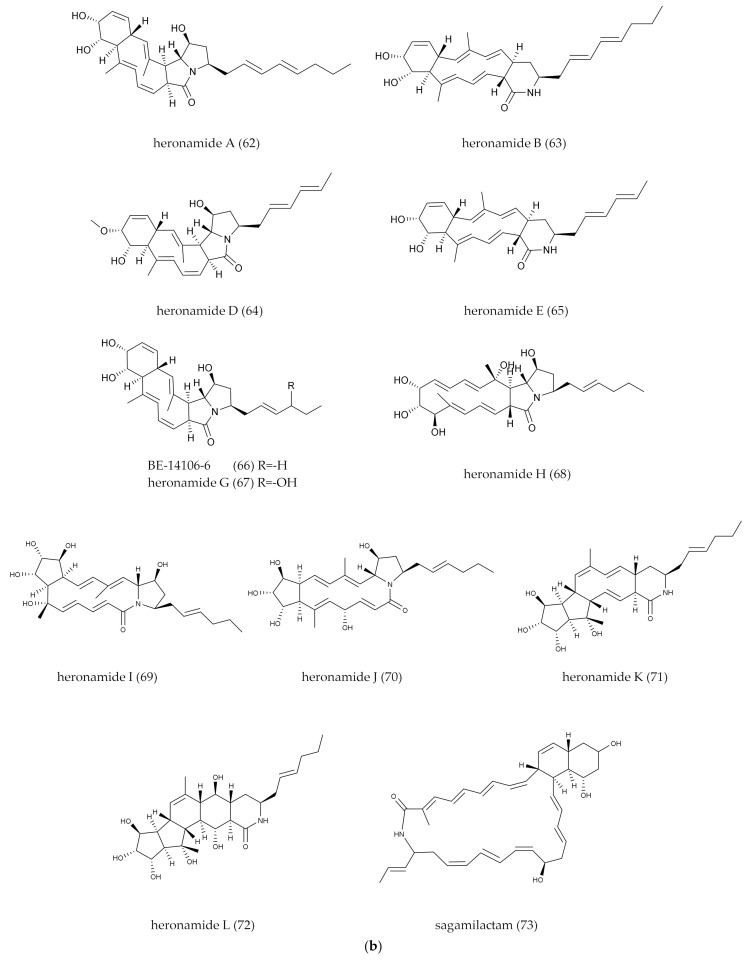

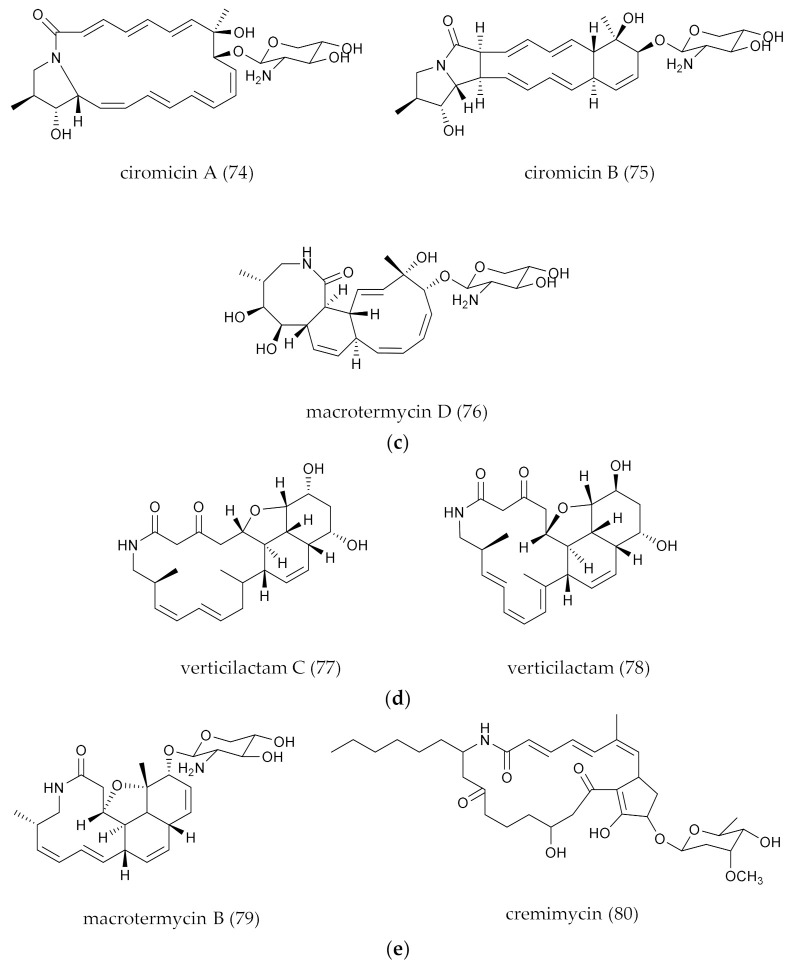
(**a**) Polycyclic PMLs (the macro-ring of PMLs generates an intramolecular polycyclic system through intramolecular addition reactions.). (**b**) Polycyclic PMLs with fatty acid chain. (**c**) Polycyclic PMLs with glycosylated groups. (**d**) Polycyclic PMLs with intramolecular epoxidation groups. (**e**) Polycyclic PMLs with sugar, epoxidation or five-member ring groups.

**Figure 3:**
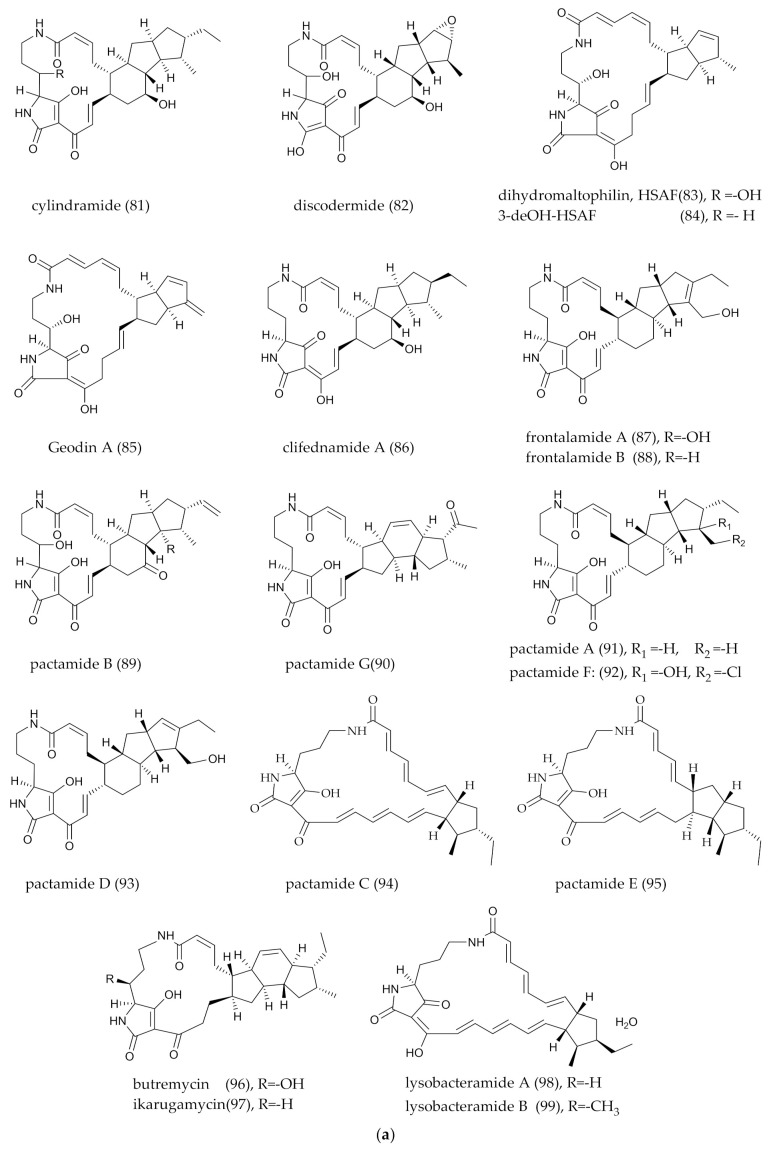

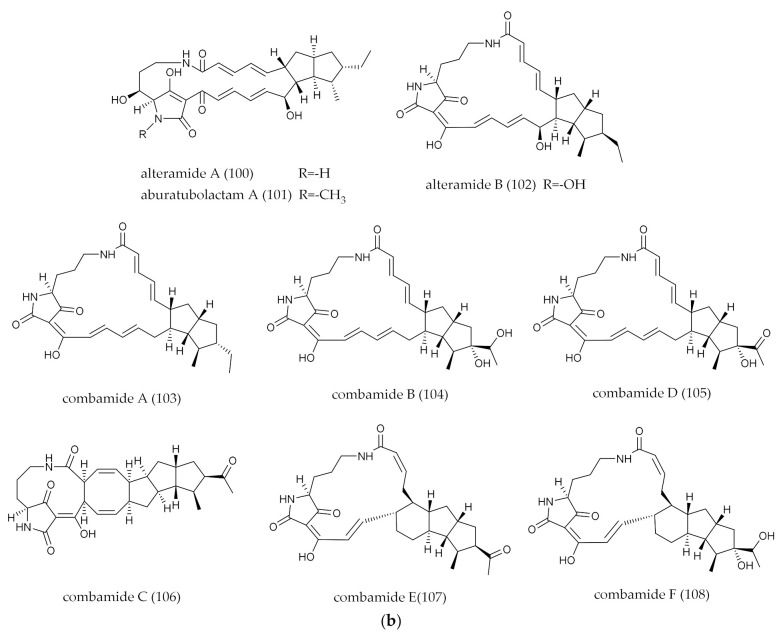
(**a**) Polycyclic tetramate macrolactams (PTMs), a branch of polycyclic PMLs. (**b**) Polycyclic tetramate macrolactams (PTMs), a branch of polycyclic PMLs.

The structure of PMLs is characterized by a ubiquitous 16–34 membered lactam ring with numerous double bonds. Hydrophilic groups, particularly the amide groups, are located in the closed ring of the carbon skeleton. There must be at least three olefinic bonds on the primary carbon skeleton. Five distinctive secondary structural features, including a ring with hydroxyl and methyl groups, the hydrophobic structure with hydrocarbon chains and a possible linkage to a glycoside, intramolecular epoxidation and intramolecular polycyclic structure. Despite the resources of PMLs being diverse (marine, sea island, forest soil, insects and plants), the marine-derived PMLs are gradually increasing in recent years, indicating that the marine environment is a non-negligible space for PMLs mining. According to the features of the PMLs’ structures from 1972 to 2021, we classified them into two major groups, mono- and polycyclic, and then subdivide them into smaller branches that contain aliphatic chains, sugar groups and epoxidation groups. Among all PMLs, polycyclic tetramate macrolactams (PTMs) are different from others in the initial pathway, so they were classified as a separate branch in the polycyclic group. The detailed information on PMLs’ structures, bioactivities and producers was shown in Figure 1a–e, Figure 2a–e and Figure 3a,b) and Table 1.

**Table 1:** The structure, producing strain, resources, biological activity and access strategy information of polyene macrolactams.

NO	Compound	Character	Member	Strain	Resource	Biological Activity	Access Strategy	Ref.
1	cyclamenol A	Mono-ring	20	*Streptomyces* sp. MHW 846	Default	Cytotoxicity, Anti-inflammatory	Natural screening and isolation	[13]
2–6	kenalactams A–E	Mono-ring	22–32	*Nocardiopsis* sp. CG3(DSM 106572)	Saltpan	C-E Cytotoxicity antibacterial; anti-viral (HCV); C-antifungal,	Bioassay-guided metabolomic analyses	[20]
7	micromonosporin A	Mono-ring	24	*Micromonospora* sp.	Peat swamp	Unable to be evaluated	Natural screening and isolation	[21]
8–9	agA and agB	Mono-ring	24	*Streptomyces* sp. CS149 mutants	Insects	Cytotoxicity	Combinatorial Biosynthesis and Mutasynthesis	[22,23]
10	FW05328-1	Mono-ring	26	*Micromonospora* sp.	Marine	Cytotoxicity	Natural screening and isolation	[24]
11	bombyxamycin A	Mono-ring	26	*Streptomyces* sp. SD53	Insects	Antibacterial and Cytotoxicity	Insect-bacterial mutualisms	[4]
12	bombyxamycin C	Mono-ring	26	*Streptomyces* sp.	Insects	Antibacterial	Insect-bacterial mutualisms	[25]
13–15	lobosamide A/B/C	Mono-ring	26	*Micromonospora* sp. RL09-050-HVF-A	Marine	Anti-trypanosomal	Genomic mining	[10]
16	micromonolactam	Mono-ring	26	*Micromonospora* sp. CMS I2-32	Marine	No activity to bacterial and fungal	Chemical screening (LC-DAD-ESIMS)	[26]
17	sceliphrolactam	Mono-ring	26	*Streptomyces caementarium*	Mud	Anti-fungal	Insect-bacterial mutualisms	[27,28]
18–19	mirilactams A/B	Mono-ring	26	*Micromonospora* sp. RL09-050-HVF,*Actinosynnema mirum*	Marine	Lack of anti-trypanosomal activity	Natural screening and isolation	[10,29]
20	salinilactam A	Mono-ring	26	*Salinispora tropica*	Marine	Default	Genome-Guided Discovery	[30]
21	streptolactam A	Mono-ring	26	*Streptomyces* sp. OUCMDZ-3159	Marine	Antifungal	Natural screening and isolation	[31]
22	BE-14106 (GT32-A)	Mono-ring/fatty acidchain	20	*Streptomyces* sp	Sea island soil	Antibacterial, Cytotoxicity	Natural screening and isolation	[5,32]
23	GT-32 B	Mono-ring/fatty acidchain	20	*Streptomyces* sp	Soil	Weak antibacterial, Cytotoxicity	Natural screening and isolation	[33]
24	JBIR-150	Mono-ring/fatty acidchain	20	*Streptomyces* sp.	Marine	Cytotoxicity	Bioassay-guided and chemical screening (LC-DAD-ESIMS)	[34]
25	heronamides C	Mono-ring/fatty acidchain	20	*Streptomyces* sp.	Marine	Non-cytotoxic, Effect on cell morphology	Chemical screening (LC-DAD-ESIMS)	[35]
26	8-deoxyheronamide C	Mono-ring/fatty acidchain	20	*Streptomyces* sp.	Marine	Non-cytotoxic, target membrane	Bioassay-guided	[36]
27	heronamides F	Mono-ring/fatty acid chain	20	*Streptomyces* sp. SCSIO 03032	Marine	Cytotoxicity	Chemical screening (LC-DAD-ESIMS)	[37]
28	ML449	Mono-ring/fatty acidchain	20	*Streptomyces* MP39-85	Marine	Antibacterial, Cytotoxicity	Bioassay-guided	[6]
29	aureoverticillactam	Mono-ring/fatty acidchain	22	S*treptomyces aureoverticillatus* (NPS001583)	Marine	Antibacterial, Cytotoxicity	Bioassay-guided	[38]
30–31	macrotermycins A/C	Mono-ring/Glycosylation	20	Amycolatopsis sp. M39	Insects	Anti-bacterial and antifungal parasite	Bioassay-guided metabolomic analyses	[39]
32–33	vicenistatin/vicenistatin M	Mono-ring/Glycosylation	20	*Streptomyces halstedii* HC34	Sea island soil	Antitumor or Against xenografted models	Natural screening and isolation	[8]
34	sannastatin	Mono-ring/Glycosylation	20	*Streptomyces sannanensis*	Animal waste	Cytotoxicity	Natural screening and isolation	[40]
35	incednine	Mono-ring/Glycosylation	24	*Streptomyces* sp. ML694-90F3	Default	Inhibit anti-apoptotic	Natural screening and isolation	[12]
36	silvalactam	Mono-ring/Glycosylation	24	*Streptomyces sp.*	Plant	Anti-G^+^ bacteria, Cytotoxic activity	Natural screening and isolation	[41]
37	auroramycin	Mono-ring/Glycosylation	24	*Streptomyces roseoporous* NRRL 15998	Default	Anti-fungal, anti-MASA	CRISPR-Cas mediated genome editing	[7]
38–39	sipanmycins A and B	Mono-ring/Glycosylation	24	*Streptomyces* sp. CS149	Insects	Cytotoxicity	Combinatorial biosynthesis and mutasynthesis	[23]
40	mirilactam E	Mono-ring/epoxidation	26	*Actinosynnnema mirum* NBRC 14,064	Plant	Not antibacterial activity and cytotoxicity	Co-culture	[29]
41	mirilactam D	Mono-ring/epoxidation	26	*Actinosynnnema mirum* NBRC 14,064	Plant	No antibacterial activity and cytotoxicity	Co-culture	[29]
42	bombyxamycin B	Mono-ring/epoxidation	26	*Streptomyces* sp. SD53	Insects	Antibacterial, cytotoxicity	Insect-bacterial mutualisms	[4]
43	dracolactam B	Mono-ring/epoxidation	26	*Micromonospora wenchangensis* HEK-797	Marine	Default	Co-culture	[42]
44	pretilactam	Mono-ring/epoxidation	26	*Actinosynnema pretiosum* ATCC 31565	Default	No anti-bacterial (Bs and Ca)	Genome-Guided Discovery	[43]
45	streptolactam C	Mono-ring/epoxidation	26	*Streptomyces* sp. OUCMDZ-3159	Marine	Antifungal (Ca)	Natural screening and isolation	
46–47	cyclamenol E-F	Mono-ring/A five-member ring	20	*Streptomyces* sp. OUCMDZ-4348	Sand, the Antarctic	E antitumor, no cytotoxicity	Bioassay-guided	[44]
48	leinamycin	Mono-ring/A five-member ring	20	*Streptomycetes* sp. S-140, *Streptomyces atroolivaceus*	Soil	Antibacterial, antitumor	Genomic mining	[9,45,46]
49	hitachimycin/stubomycin.	Mono-ring/A five-member ring	22	*Actinomycete* strain No. KM-4927*/Streptomyces* strain No. KG-2245	Soil	Antiprotozoal/antifungal	Natural screening and isolation	[18]
50–51	niizalactam A and B	Mono-ring/A five-member ring	26	*Streptomyces* sp. NZ-6	Terrestrial	No antimicrobial activity and cytotoxicity	Co-culture	[47]
52	piceamycin	Mono-ring/A five-member ring	26	*Streptomyces* sp. SD53	Insects	Antibacterial, Cytotoxicity	Insect-bacterial mutualisms	[25]
53	viridenomycin	Mono-ring/A five-member ring	26	*Streptomyces gannmycicus*	Soil	Cytotoxicity	Natural screening and isolation	[48,49]
54–56	cyclamenol B–D	Polycyclic	20–22	*Streptomyces* sp. OUCMDZ-4348	Sand, Antarctic	Only B selective cytotoxicity	Bioassay-guided	[13]
57	dracolactam A	Polycyclic	26	*Micromonospora wenchangensis* HEK-797	Marine	Default	Co-culture	[42]
58	mirilactam C	Polycyclic	26	*Actinosynnnema mirum* NBRC 14,064	Plant	No antibacterial activity and cytotoxicity	Co-culture	[29]
59	verticlactam B	Polycyclic	24	*Streptomyces avermitilis* SUKA17*/Streptomyces spiroverticillatus* JC-8444	Default	Anti-parasitic	Metabonomics LC/MS	[50]
60	streptolactam B	Polycyclic	26	*Streptomyces* sp. OUCMDZ-3159	Marine	No prominent cytotoxic activity	Natural screening and isolation	[31]
61	tripartilactam	Polycyclic	26	*Streptomyces* strain SNA112	Insects	Na^+^/K^+^ATPase inhibitor	Insect-bacterial mutual and Chemical screening (LC-DAD-UV)	[51]
61	niizalactam C	Polycyclic	26	*Streptomyces* sp. NZ-6	Terrestrial	No antimicrobial activity and cytotoxicity	Co-culture	[47]
62–63	heronamides A/B	Polycyclic/fatty acid chain	28	*Streptomyces* sp. CMB-M0406	Marine	No antibacterial activity and cytotoxicity	Chemical screening (LC-DAD-ESIMS)	[35]
64–65	heronamides D–E	Polycyclic/fatty acid chain	28	*Streptomyces* sp. SCSIO 03032	Marine	No antibacterial activity and cytotoxicity	OSMC (alternative medium)	[37]
67–72	heronamides G–L	Polycyclic/fatty acid chain	28	*Streptomyces niveus*	Forest soil	No antibacterial activity and cytotoxicity	Bioassay-guided	[52]
73	sagamilactam	Polycyclic/fatty acid chain	34	*Actinomadura* sp. K13-0306	Sea island soil	Anti-trypanosomal	Chemical screening (LC-DAD-ESIMS)	[11]
74–75	ciromicins A–B	Polycyclic/glycosylation	22	*Nocardiopsis* sp. FU40	Default	Cytotoxicity	Co-culture and metabolomic	[53]
76	macrotermycin D	Polycyclic/glycosylation	20	*Amycolatopsis* sp. M39	Insects	No antibacterial and antifungal activity	Bioassay-guided metabolomic	[39]
77	verticilactam C	Polycyclic/epoxidation	24	*Streptomyces avermitilis* SUKA17	Default	Anti- malaria parasite	Heterologous expression	[50]
78	verticilactam	Polycyclic/epoxidation	24	*Streptomyces spiroverticillatus* JC-8444	Default	No biological effects	Chemical screening (LC-DAD-ESIMS)	[54]
79	macrotermycins B	Polycyclic/glycosylation and Epoxidation	20	*Amycolatopsis* sp. M39	Insects	No antibacterial and antifungal activity	Bioassay-guided metabolomic analyses	[39]
80	cremimycin	Polycyclic/fatty acid chain/glycosylation	22	*Streptomyces* sp. MJ635-86F5	Soil	Anti-G ^+^ bacterial, Cytotoxicity	Natural screening and isolation	[55]
81	cylindramide	Polycyclic/PTM	26	*Halichondria cylindrata*	Marine	Cytotoxicity (B16)	Natural screening and isolation	[56]
82	discodermide	Polycyclic/PTM	26	*Discodermia dissoluta*	Marine	Cytotoxicity and antifungal	Natural screening and isolation	[57]
83	HSAF	Polycyclic/PTM	26	*Lysobacter enzymogenes*, *Streptomyces* sp. SR107	Soil	Anti-fungal	Bioassay-guided	[58,59]
84	3-deOH-HSAF	Polycyclic/PTM	26	*Lysobacter enzymogenes*	Soil	Lost antifungal activity	Gene knockout	[60]
85	geodin A	Polycyclic/PTM	26	Geodia	Marine	Cytotoxicity	Bioassaydirected	[61]
86	clifednamide A	Polycyclic/PTM	26	*Streptomyces* sp. strain JV178	Garden soil	Default	Combinatorial biosynthesis	[62]
87–88	frontalamides A–B	Polycyclic/PTM	26	*Streptomyces* sp. SPB78	Marine	Antifungal	OSMAC + chemical screening	[63]
89–95	pactamide A--G	Polycyclic/PTM	26	*Streptomyces pactum* SCSIO 02999	Default	Cytotoxicity	Promoter engineering and heterologous expression	[64]
96	butremycin	Polycyclic/PTM	26	*Micromonospora* sp. K310	Mangrove	Weak antibacterial	LC- MS	[65]
97	ikarugamycin	Polycyclic/PTM	26	*Streptomyces* sp. NO 8603	Soil	Antiprotozoal, anti-G^+^ bacterial	Natural screening and isolation	[66]
98–99	lysobacteramide A and B	Polycyclic/PTM	26	*Lysobacter enzymogenes* C3	Default	Cytotoxicity; B- Anti-fungal	OSMC	[67]
100	alteramide A	Polycyclic/PTM	26	*Alteromonas* sp.	Marine	Cytotoxicity	Natural screening and isolation	[68]
101	aburatubolactam A	Polycyclic/PTM	26	*Streptomyces* sp. SCRC A-20	Marine	Inhibit superoxide anion generation	Default	[69]
102	alteramide B	Polycyclic/PTM	26	*Lysobacter enzymogenes* C3	Default	Anti-fungal(yeast and Ca)	Natural screening and isolation	[70]
103–108	combamides A−F	Polycyclic/PTM	26	*Streptomyces* sp. S10	Garden soil	F weakly inhibited SPI-1	Combinatorial Biosynthesis	[71]

NO—corresponding to the serial number of the structure in Figure 1, Figure 2 and Figure 3. Member—means how many atoms are in the single-closed ring structure. Default means not mentioned in the original reference.

## 3. PMLs Production Strategies

**Figure 4:**
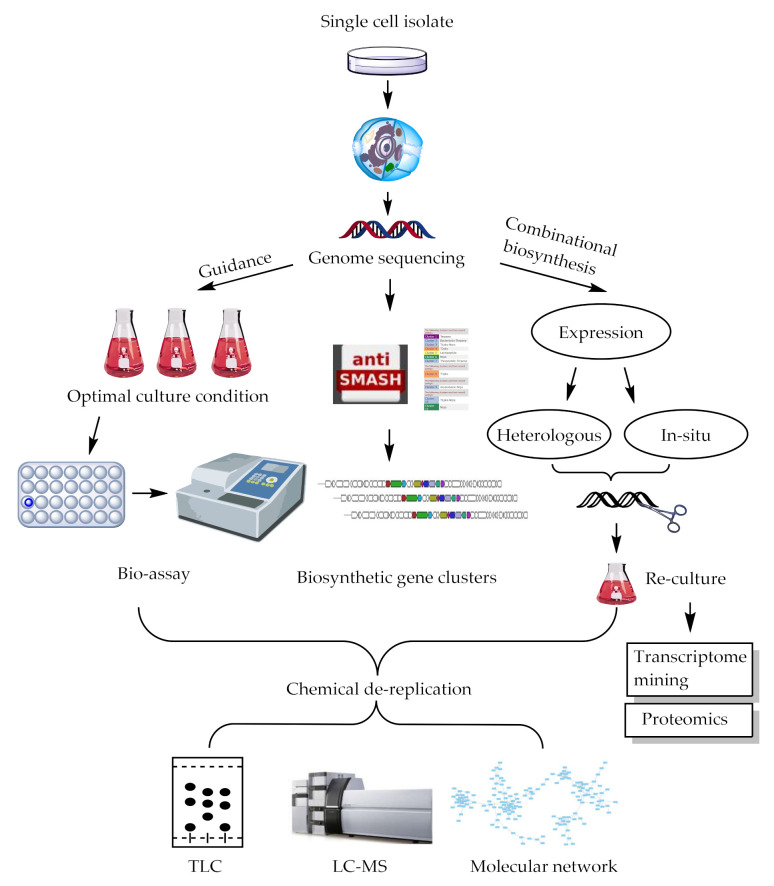
An overview of discovery strategy of natural products.

For years, scientists devoted considerable efforts to the discovery of novel bioactive compounds in the hope of finding drug leads or drug candidates. However, the frequent rediscovery of known natural compounds has become increasingly common and chemists squandered far too much attention on repetitive screening. Seeking new microorganisms for a new type of chemical scaffolds has proven to be effective [72,73]. In addition to genomic mining for microorganism de-replication, chemical informatics methods are also critical. Moreover, scientists have developed several combinatorial biosynthetic techniques to combine with complicated metabolic networks, largely in *Streptomyces*, obtaining a variety of natural compounds, including PMLs. A schematic of these strategies was shown in Figure 4.

### 3.1. Culture Strategies for PMLs Production

#### 3.1.1. Elicitors Supplementary

In addition to the genetic-engineering-oriented combinative biosynthesis method, the traditional simple and inexpensive method of adding elicitors [74], also known as allelochemical such as sceptrin [75], γ-butyrolactone [76,77,78], methyl-lenomycin furan and D-homoserine lactones (HSLs) synthesized by Gram-negative bacteria [79] were used to increase the diversity of chemical structures of secondary metabolites thereof. Macroporous resins were added into the culture medium to manipulate the inhibition of transcription feedback effect in the synthesis of secondary metabolites [80]. Previous studies demonstrated that the presence of elicitors can effectively activate the expression of silenced gene clusters and enhance the expression of the functional gene [81]. The methodology of supplementing growth media with elicitors was usually applied in combination with genetic manipulation to optimize culture conditions.

#### 3.1.2. Bacterial Co-Cultivation

In some cases, silent or cryptic BGCs (Biosynthetic Gene Clusters) in a microorganism could be activated by the interaction with other microbial species. Compared to genetic manipulations, the co-incubation method is a more promising and effective method to induce the production of novel secondary metabolites [82], which may expand the chemical diversity of the second metabolites of microorganisms.

Mirilactams A–E (**18**–**19**, **58**, **40**–**41**) [10,29], dracolactams A–B (**57**, **43**) [42] and niizalactams A–C (**50–51**,**61**) [47] are natural products produced by co-cultured with the mycolic acid-containing bacterium (*Tsukamurella pulmonis*). *T. pulmonis* tends to produce new intramolecular polycyclic compounds lacking antibacterial activities, cytotoxicities and anti-trypanosomal, activities as shown in Table 1. However, from the available data, for the activities of intramolecular polycyclic PMLs shown in Table 1, it is noticeable that some of intramolecular polycyclic PMLs analogues, including frontalamides (**87**–**88**) [63], ikaugamycin (**97**) [66], are shown to possess antifungal and anti-protozoal activities. In contrast to many other published literature, this is no special phenomenon in nature, and these compounds showed ecological significance. We hypothesized that the mycolic acid-containing bacterium may reduce the side-effects of these compounds on their survival, for example, secreting the corresponding enzyme to modify the compound to decrease toxicity. However, in effect, adding mirilactam A to the medium of mycolic acid-containing bacteria has not led to the appearance of mirilactam C–E (**58**, **40**–**41**) [29]. This result demonstrated that mirilactam C–E (**58**, **40–41**) was not produced by biotransformation from mirilactam A, and the combination culture probably induced the function of specific enzymes in the process of epoxidation and cyclization in the gene cluster of mirilactam C–E substance biosynthesis.

Interestingly, mirilactams C–E (**58**, **40**–**41**) and a series of typical intramolecular polycyclic compounds produced by *Streptomyces* in pure culture did not show any antimicrobial activities. Heronamides (**25**–**27**, **62**–**72**) and heronamide C (**25**) showed notable morphologically reversible non-cytotoxic effects on mammalian cells. Deoxyheronamide C also displayed cell membrane-associated bioactivities. Apart from the above two compounds from the family of heronamides, no other heronamides exhibited cytotoxicity and antibacterial activity [35,37,52,83]. These results do not conform with the rules of nature due to the fact that bacteria produced these useless compounds involving large metabolic costs in terms of energy and nutrients. By using Hes1-immobilized beads, iso-micromonolactam was a possible activator of neural stem cells, implying the potentially latent function of PMLs [84]. The underlying cause of this phenomenon deserves an in-depth investigation chemically and biologically in the future.

### 3.2. Chemical Analytical De-Replication

The biological taxonomic de-duplication method was based in the comparison of 16S rDNA and the analysis of morphology. Due to the similarity of biological morphology and the fact that 16S rDNA can only be identified at the genus level, many microbial species containing different secondary metabolite biosynthetic gene clusters with slight differences in morphology and 16S rDNA sequences would be overlooked. Due to the fact that a large number valuable isolates were neglected by the means of taxonomic deduplication, chemical deduplication is especially important. Remarkably, many studies used the genomic prescience of natural products to choose the prioritization of feature response data for further isolation. A wide variety of statistical and analytical techniques based on algorithms of machine learning combined with visualized response map was applied to improve prioritization, such as global natural products social molecular networking (GNPS) and MS-based visualizing techniques [85,86,87]. With the assistance of genomic analysis [88] and predicting potential drug candidates from organisms and monitoring metabolites and mapping analysis of metabolomics via liquid chromatography-mass spectrometry (LC-MS)/MS [53,72], the known metabolites were identified and excluded. Thus, the hunting zone for novel substances was effectively narrowed, whereby macrotermycins A–D (**30**–**31**,**76**,**79**) [39], aplysiatoxin homologues [89] and amicoumacin antibiotics [90] were discovered from a complex secondary metabolic profile. Another metabolomic approach for de-replication was achieved by employing nuclear Magnetic Resonance hyphenated with liquid chromatography (LC-NMR). With the continuous expansion of the NMR spectra database, analyzing NMR spectra by artificial intelligence algorithm, for example, the star “DP4-AI” [91], will hopefully replace manual analysis in the future. This method can realize the rapid identification of trace compounds and improve the accuracy thereof.

### 3.3. Genetic Tools for PMLs Biosynthesis

#### 3.3.1. Genomic Mining

One of the traditional genomic mining methods for obtaining novel bioactive natural products was based on the isolation of rare actinomycetes from neglected environments. In this process, 16S rRNA gene sequencing was applied for preliminary microbial identification and de-replication [92]. However, although nucleic acid sequence homology reached 99%, it was not certain that the strain can produce the same secondary metabolites because it contained different functional genes [93], which induces difficulties for 16S rRNA gene-dependent genomic mining.

In recent years, most PMLs were obtained from actinomycetes by another genomic mining method. The method was used to screen rarely related key genes and the fragment of synthase genes as signature probes. It was also applied in whole genome sequence (WGS) scanning [94]. WGS, a powerful tool, is used to predict the types of microbial secondary metabolites, but somehow the costs of this method are still high, particularly in the High-Through Screening (HTS) process.

The formation of small artificial meta-genomics may be an economically viable approach, involving meta-genome sequencing, splicing, and restoring the individual draft genome sequence from a different genus. Firstly, around three or more mixed strains with a wide variety of genera were selected based on 16S rRNA gene sequence to create a direct small metagenome. Then, the results of the metagenomic sequencing artificial combination were spliced and assembled completely according to the whole genome sequence of the corresponding strains in the NCBI database. Lastly, the functional gene clusters associated with secondary metabolites were found and identified. The key to this approach is reliance on bioinformatics tools of how to assemble the genome sequencing results from the directed construction of small metagenomes and assign them to each strain accurately. The discovery-based approach combined with biosynthetic gene analysis complements the knowledge-based approach by exploring the vast combinatorial biosynthesis repertoire found in nature [94].

#### 3.3.2. Heterologous or In Situ Expression and Synthetic Biology

Historically, obtaining secondary metabolites by expressing genomic modules in the heterologous hosts was a common approach. However, many secondary metabolites are difficult to biosynthesize by this method because the lack of specific precursors, suitable alien promoters, biosynthetic functional protein in the host and the display of toxicity to hosts. In 2011, Gomez-Escribano and Bibb [81] proposed an innovative idea that constructs a background “clean” expression host, *Streptomyces coelicolor* M145, to improve the production of metabolites transcribed by alien biosynthetic gene clusters. From then on, more and more evidence showed that the engineered *S. coelicolor* host without native BGCs was appropriate for the heterologous expression of a wide spectrum of BGCs [95,96], which significantly and exponentially increased the yields of natural products, such as mithramycin A [97]. The methods mentioned before are summarized as follows: combined bacterial or fungal genome scanning, whole-genome sequencing and comparative genomics analysis technology to determine some specific biosynthetic gene clusters. The identification and confirmation of the products produced by heterologous expression in engineered bacterial hosts were difficult to a certain extent [7]. Nevertheless, cloning the BGCs larger than 100 kb in length is still a challenge in heterologous expressions [98].

To enhance the production of PMLs by heterologous expression, a strong constitutive promoter such as PermE* is often added to the biosynthetic cluster, and negative expression regulatory cassettes are always deleted during genomic manipulation [62]. These strategies have made a successful biosynthesis of new PTMs by introducing BGCs into *S.lividans* [99].

Actinomycetes generally contain many silenced groups, which belong to the secondary metabolite generation branch pathway, and can only be activated in some special facts or conditions. For instance, in 2019, Lim et al. successfully obtained auroramycin with the novel structural potent antibiotic family of PMLs from *Streptomyces roseosporus* via activating the biosynthesis pathway by CRISPR-Cas9 mutation technology [7].

**Figure 5:**
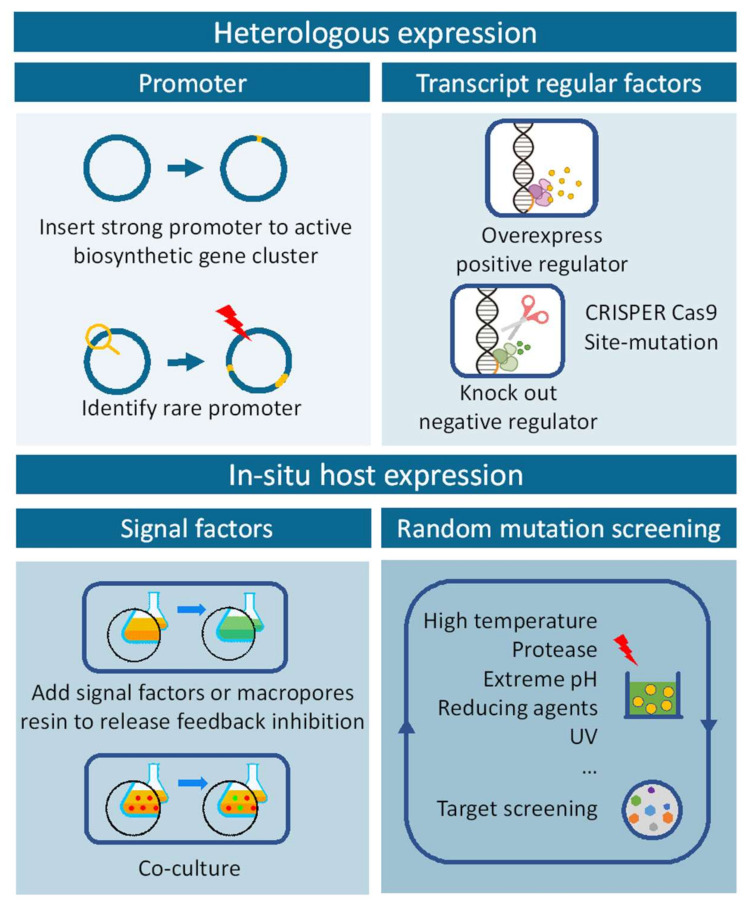
The workflow for discovery of new compounds in heterologous or in situ hosts expressing.

In addition, although the inactivated endogenous biosynthetic pathways of various engineered hosts have been constructed by knockout or site mutation, the major ones were mainly still *Streptomyces*. There were a few reports on the construction of *Micromonospora* and other engineered hosts for target BGCs expression. Therefore, with more and more novel biosynthetic genomic clusters explored and revealed, the development of various rational types of hosts is in urgent need of research. The process described in the above was illustrated in a schematic of Figure 5.

Synthetic biology is a theoretically feasible method that exchanges promoters, SD sequences, ORFs and terminators and assembling them into new biological systems such as novel metabolic pathways to explore rare secondary metabolites. All existing combinatorial biosynthetic strategies were mapped out based on the biosynthetic assembly line: that is, proteins of known function corresponding to their encoding genes, manipulation of which affords the engineered biosynthetic pathways that produce the directed natural products. The typical merit of the knowledge-based combinatorial biosynthesis method is that it modifies the targeted functional moiety and keeps the rest of the scaffold unchanged. For instance, overexpressing a transcriptional activator leads to the discovery of largimycins from *Streptomyces* in Leinamycin Family [99].

With the understanding of each component gene scaffold in controlling the biosynthesis pathway of natural products, the modular functional protein of different microbials would be recombined into a new natural product assembly line, which might artificially obtain novel compounds by using microbial BGCs.

#### 3.3.3. Chemical Synthesis under the Guidance of Bioinformatics

In 2022, Lei Li et al. combined sequence-based metagenomic mining with bio-informatical analysis to identify and predicted six natural active menaquinone-binding (MK binding) antibiotics (MBAs) [100]. Moreover, chemical synthesis was creatively applied to obtain the six predicted MBAs instead of synthesis by microorganisms in vivo. The results of this study suggested that the approach might be applicable to access novel PMLs in the future.

## 4. PMLs Biosynthetic Pathways

The strategies for the discovery of natural products of microbial origin are usually based on detailed information on the biosynthetic pathways of the corresponding substances. Therefore, after recognizing the structures of new natural products, it is imperative to acquire knowledge of their biosynthetic pathways in vitro and in vivo.

PMLs have five main structural features, including a ring skeleton with hydroxyls and methyl groups, hydrocarbon chains, a possible linkage to a glycoside, intramolecular epoxidation and intramolecular polycyclic structure.

PMLs’ structural diversity and diverse biological activities were largely due to a variety of non-proteinogenic β-amino acids in their polyketide starter units, different PKS-I synthetic enzyme modular compositions involving ketone synthase (KS), acyl-transferase (AT), acyl carrier protein (ACP), ketone reductase (KR), dehydrogenase (DH), thioesterase (TE) and specific post-PKS PMLs made by combining polyketide synthase (PKS-I) and non-ribosomal polypeptide synthase (NRPS) as well as polyketide reductase (KR). Condensation–adenylation–thiolation (C-A-PCP) elements were included in the NRPS minimal module. To produce the above mature products, precursor substances discharged from multi-enzymatic assembly lines might be subjected to one or more decoration courses of acylations, alkylations, hydroxylations, glycosylations, hetrocyclizations and intermolecular cyclizations. Antibiotic action normally necessitates these post-modifications and tailoring.

The biosynthesis processes of the compounds would be narrated by separating them into the following four phases based on the structural properties of the aforementioned PMLs and important enzymes engaged in the biosynthetic route. The first phase was the formation of β-amino acid starter units and then transferring them into the PKS I type assembly; the second was chain extension and the formation of olefinic bonds in the ring as well as cyclization by thioesterase; the third was intramolecular epoxidation and intramolecular polycyclic formation in post-modification processes; the last was glycosylation modification. According to the structure of PMLs, not all PMLs compounds were synthesized through the four complete processes mentioned above. PTMs have different biosynthetic pathways from the corresponding polyenic amino acid, which were illustrated in detail by Guangtao Zhang et al. [101].

### 4.1. PMLs Initial β-Amino Acid Units

**Figure 6:**
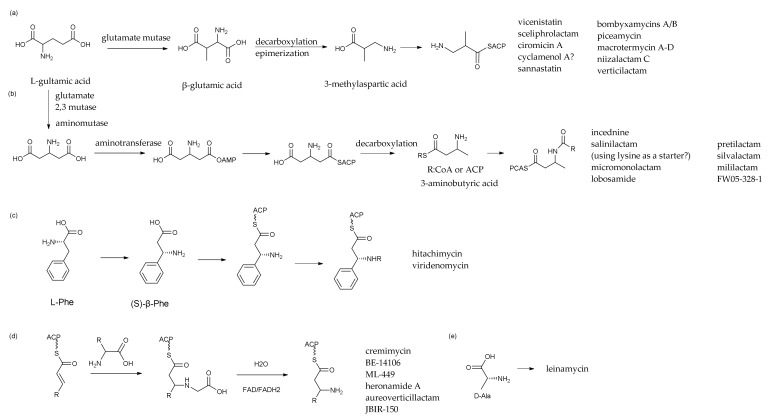
The comparison and analysis of PMLs’ biosynthetic starter units. (**a**–**e**) represent five different β-amino acid initiation modes.

Brandon I. Morinaka et al. evaluated the data of bacterial meta-genomes deposited in the Genebank database and found that a variety of microorganisms may produce β-amino acid-containing products [102]. The structural variety of macrolactam polyketides is generated by the presence of diverse β-amino acid starting units in their polyketide skeletons because amino groups of amino acids are essential to the formation of their structures. In addition, incorporating these β-amino acids into their polyketide skeletons was a key process. Five types of amino acid initiation units of related compounds were summarized in Figure 6 by a comparative analysis of the structures of the six types of known polyene macrocyclic lactams and comparing the relevant literature on the biosynthetic pathway of macrolactams [103]. Thus far, several β-amino acid starting units in the biosynthesis of macrolactam polyketides have been revealed, as has the process for selective incorporation [104].

These aminomutase, decarboxylase, aminotransferase and acyltransferase were involved in the biosynthesis of β-amino acid units. There include glutamate amino-mutase, lysine amino-mutase, arginine amino-mutase, S-adenosyl-l-methionine (SAM) motif and pyridoxal 5′-phosphate (PLP)-binding motif in the family of amino-mutases. More and more studies focused on the functional genes encoding amino-mutase were conducted, which would shed light on finding new amino mutases that can trace back to the specific structural units in the future.

The first mode of synthesis for β-amino acid starter units involved in PMLs, which was represented for the biosynthetic of vicenistatin (**32**) [105,106,107], β- glutamate derived from L-glutamic acid by the action of glutamate amino-mutase-realized intramolecular rearrangement of the α-amino group to the β-position, was decarboxylation and epimerization to 3-Methylaspartate (Figure 6). For instance, 3-MeAsp needed to be decarboxylated to 3-aminoisobutyrate before being accepted by the ACP module and entering the phase of PKS I-type carbon chain extension. By investigating the compound structures and the BGCs gene cluster, we summarized and proposed that, except for 3-MeAsp, there are many other PMLs implicated in this metabolic route, such as sceliphrolactam (**17**), ciromicin A (**74**), sannastatin (**34**), bombyxamycin A (**11**), piceamycin (**52**), macrotermycin A–D (**30**–**31**,**76**,**79**), niizalactam C (**58**), verticilactam (**59**) and so on.

To confirm the beginning unit of the amino acid involved in the biosynthesis route of kenalactam A (**2**), 11 amino acids were added separately into the medium, and then the products were characterized using the LC-MS technique by Omar Messaoudi et al. (2018). As a consequence, L-alanine is likely to be the first unit in the structure of kenalactam A [20] because the amide groups in kenalactam A are separated by one carbon from the methyl branched chain at C24, which may mean that 3-methyl aspartic acid is the starting unit of amino acid for biosynthesis.

The result that contradicts the aforementioned authors’ experimental findings were deduced from the reported synthesis pathway of β-amino acid analogues such as vicenistatin (**32**), sceliphrolactam (**17**) [28] and so on. In addition, although β-amino acid is the beginning unit of amino acid for biosynthesis, L-alanine could be a protecting group for avoiding self-cyclization and for connecting to the starter unit and eventually integrating into the PKS I-type biosynthetic pathway of kenalactam A (**2**) via Michael addition reactions [104]. Additional research is needed to support this hypothesis.

The second mode represented for incednine (**35**) biosynthesis [108,109,110,111], β-glutamate derived from α-L-glutamate by the action of glutamate 2,3-aminomutase is decarboxylated to (S)-β-aminobutyrate by the PLP-dependent β-glutamate decarboxylase IdnL3 (Figure 6). Then, the mentioned β-amino acid combined with acyltransferase (AT module) constructed a β-keto acyl intermediate. Michael’s addition to an α,β-unsaturated acyl intermediate in the polyketide pathway is also involved. By analyzing the enzyme function and the result of the amino acid site-mutation replacement experiment, it was found that the adenylation enzyme in synthesis was likely to be the substrate of the β-amino acid starter unit. Many types of research revealed that the different amino acid residues in the 220th bit can affect the activity of the IdnlL enzyme, and the specificity of the substrate is determined by the function of the enzyme [109].

A set of glutamate 2,3-aminomutase and β-glutamate decarboxylase gene sequences was found in actinomyces, which can produce salinilactam A (**20**) [30], micromonolactam (**16**) [26], lobosamide(**13**–**15**) [10], pretilactam (**44**) [43], silvalactam (**36**) [41] and mirilactam, suggesting that β-aminobutyrate may be the starter unit in biosynthetic pathways of these polyketides. However, the results of the isotope amino acid feeding experiment were not always consistent with the consequence of biosynthetic gene clusters such as in the case of salinilactam A (**20**). Previous results indicated two possibilities for the initial amino acid of FW05-328-1. The first possibility was that L-glutamic acid was converted to β-glutamate by aminotransferase, and then decarboxylation and epimerization were occurred in the catalysis of the various modularized enzyme to produce 3-methyl aspartic acid or 3-amino butyrate, which would enter the PKS I-type biosynthesis pathway as an amino initiating unit. Another possibility was that FW05-328-1 had a different carbon skeleton by processing a methyl group at the α position, which revealed that it may start with an α-amino acid unit rather than a β-amino acid unit. Since the above processes have not been experimentally validated, more experiments will be needed to fill the gaps.

Hitachimycin (**49**) is a representative PMLs compound containing a five-membered ring. Compared with others, hitachimycin is a macrolactam with β-phenylalanine as the starter position of its polyketide skeleton. Its biosynthesis pathway was through the characteristic of L-phenylalanine for initial amino acids, 2,3-aminomutase, making it change into β-phenylalanine, and then through modular type I-PKS biosynthesis, which contains five PKS synthetases and four amino acid carrier enzymes, a characteristic of the amidohydrolase for completing the synthesis of the compounds. Moreover, the characteristic intramolecular five-membered ring by Michael addition or a Nazarov cyclization mechanism for formation is present [112].

The cyclo-pentyl group was easily assembled in the biosynthetic process through enzyme factories and spontaneous reactions. The chemical total synthetic steps of the cyclo-pentyl group were reduced from eleven to five by chemists in eight years from 2000 to 2008 [49,113,114,115].

In addition, although the compounds such as cremimycin (**80**) [116], BE14106 (**22**) [32], ML449 (**28**) [6], JBIR150 (**24**) [34] and aureoverticillactam (**29**) [38] had different medium-chain β-amino acid starter units, they had similar amino acid incorporation mechanism in the PKS biosynthetic pathway.

Cremimycin (**80**) is a representative PMLs since the structural unit of polyene macrolactam, medium-length fatty chain and the five-membered ring was located in the structure at the same time. For instance, 3-amino nonanoate derived from glycine was combined with the β-position of a non-2-enoic acid thioester by Michael in cremimycin [116]. Thirty-three open-reading frames (ORF) were involved in the biosynthetic gene cluster, which encoded eight polyketide synthases, six deoxysugar enzymes and a characteristic group of five β-amino acid transfer enzymes [110,117,118]. Keita Amagai et al. proposed the biosynthetic pathway of cremimycin [117] in microorganisms by gene knockout, heterologous expression and feeding precursors. In the process of polyketide biosynthesis, the major precursor of propionate extender unit was methylmalonyl-CoA the primary metabolite but succinyl-CoA was also. The most common source of succinyl-CoA came from the TCA cycle [117].

Last but not least, leinamycin (**48**) was a thiazole-type heterocyclic PMLs compound. The uncommon 1,3-dioxy-1, 2-dithioalkanes were conjugated into the macrolactam ring in the structure of Leinamycin. Experiments on the location of the LNM biosynthetic gene cluster, sequence analysis, gene knockdown and mutant screening revealed that Leinamycin was produced by an AT-less NRPS-PKS hybrid system using D-alanine as a starter [6,119]. D-amino acids were activated by the A module. LNM biosynthesis and chain extension processes were carried out by discrete A and ACP modules, with an extra NRPS and six PKS added throughout the synthesis phase (Cheng 2003). L-Cys, which catalyzed the production of the thiazole group, was the particular substrate of the *lnmI* gene [45].

In summary, the biosynthetic pathway of β-amino acid was identified and validated using a variety of genetic manipulations, including deducing genomic information, isotope amino acid addition experiments, heterologous expression of BGCs, gene knockout and structural biology and enzymology functional tests in vitro.

### 4.2. PMLs Extender Unit Incorporation and Cyclization

Processing carbon chain extenders in the biosynthetic of polyene macrolactam was performed by catalyzing PKS I enzymes, affecting the members of macrolactam and the number of olefinic bonds of macro-cyclic. The combination and arrangement of different functional modules, for instance, KS-AT-DH-ER-KR-TE, may change the intra-cyclic carbon members, the position and number of unsaturated double bonds and the introduction of methyl groups [120,121].

Specifically, the KS domain catalyzed the condensation of the extender unit to an acyl-starting substrate or a developing polyketide chain in a Claisen-type reaction. KR, DH, and ER domains convert to the resultant β-keto moiety to form a β-hydroxy group, anα, β-double bond or a completely reduced methylene [122]. During polyketide chain elongation, alkylations, hydroxylations and hetero cyclizations may occur. Finally, the extended polyketide chain was usually liberated from the PKS assembly line by a thioesterase (TE) domain-catalyzed hydrolysis or macrocyclization. It should be mentioned that during the carbon chain extension of cremimycin (**80**), the natural truncated DH-split functional region was specifically used to form hydroxyl groups [123]. The significance of this biological phenomenon was worthy of further study and discussion, which could be used as a theoretical reference for designing and establishing new PKS assembly lines in the future.

### 4.3. Epoxy Groups and Intramolecular Polycyclic Systems

**Figure 7:**
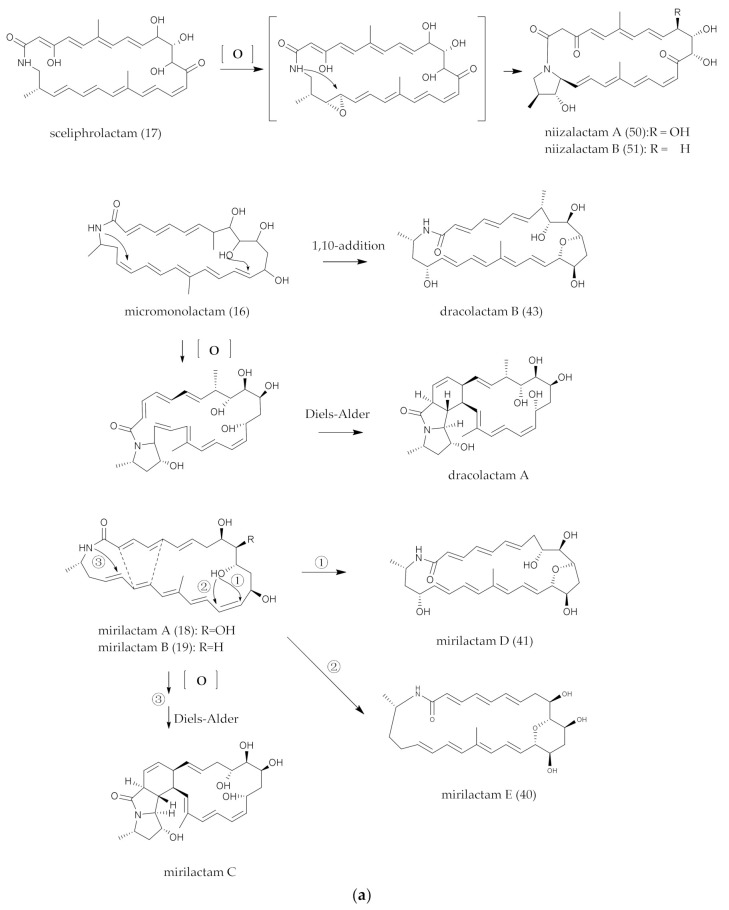

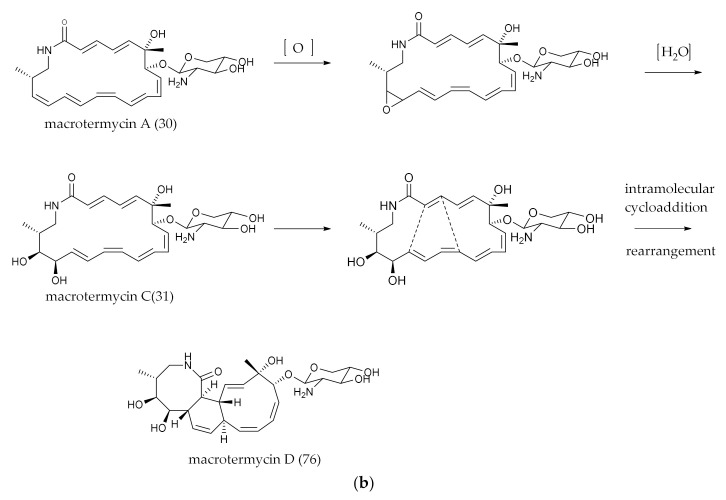
(**a**) Conjectures on the formation of intramolecular Epoxy and polycyclic groups. (**b**) Conjectures on the formation of intramolecular Epoxy and polycyclic groups.

The released polyketide intermediates were further modified by intramolecular epoxy and cyclization, which were crucial for the biological activities of the natural polyketides. From the literature, it was found that epoxy groups and intramolecular polycyclic structures formed by intramolecular oxidation and cycloaddition reactions were not unique cases in polyene macrocyclic lactam compounds. As shown in Figure 7, the processes of structural change have been predicted and represented as micromonolactam (**16**), sceliphrolactam, (**17**) mirilactam A (**18**) and B (**19**) macrotermycin A (**30**), which are accordingly conversed to niizalactam A–B (**50**, **51**), dracolactam A–B (**57**, **43**), mirilactam C–E (**58**, **40**–**41**) and macrotermycin C (**31**) and D (**76**).

In 2018, Shotaro Hoshino et al. speculated that the final epoxidized structure of mirilactam C (**58**) and D (**41**) [29] might be formed because of -NH or intramolecular nucleophilic-attacking C-11 site. This was similar to the synthesis of dracolactam A (**57**) and B (**43**) [42], according to the analysis by Schulze et al. miR-biosynthesis gene cluster and the structure and biosynthesis pathway of structural analogues. The epoxidation mechanism of mirilactam E (**40**) was different from that of dracolactam B (**43**) (Figure 7a). Mirilactam E (**40**) formed an epoxidation structure through a nucleophilic attack on C-14 by hydroxyl groups at the C-10 site. Within the biosynthetic gene cluster, miR-L was the sole enzyme that was presumed to encode the function of P450, which may interact with the hydroxyl group at the C-10 position. Studies had shown that the biosynthesis of mirilactam C–E (**58**, **40–41**) from mirilactam A (**18**) was not the result of the biotransformation of co-cultured MACA. The most likely reason was that the coexistence of MACA stimulated the cryptic P450 enzyme-producing bacteria in the biosynthesis pathway of mirilactam A (**18**) and led to intramolecular oxidation and cyclization and ultimately the generation of mirilactam C–E (**58**, **40–41**).

In brief, due to the existence of polyene bonds, there were more possibilities for intramolecular changes. The process was similar, but the mechanism may be different. For example, the [6π + 4π] [124,125] and [4π + 2π] cycloaddition reaction [122] pathways may be caused by the changes in the energy levels of the bonding and antibonding orbitals in the pericyclic reactions. Heronamides were produced via two spontaneous pathways involving alternative thermal [6π + 4π] [29] and photochemical [6π + 6π] intramolecular cycloadditions. Similarly, ciromicin B (**75**) was predicted from the product of ciromicin A (**74**) [6π + 6π] cycloaddition by photocatalytic pathway [126]. PTMs are also an important branch of PLMs [101] (Figure 3a,b), and a series of results from combinatorial biological experiments has shown that OX genes are involved in the formation of intramolecular multiple rings [127]. Wherefore, whether these reactions were formed by a non-enzymatic system (photocatalytic thermal-catalytic [36,126]) or an enzymatic system [128] was indefinite, since the enzymes associated with Diels–Alder cycloaddition reactions are rare in nature; furthermore, more experimental results are needed for elaboration.

### 4.4. Glycosylated Groups

Antibacterial, fungal and cytotoxic activities were presented in glycosylated polyene macrolactams. Sannastatin (**34**) was derived from its biosynthetic precursor vicenistatin through a selective epoxidation process in *S. Sannanensis* [40]. The sugar group of sannastatin (**34**) was the same as vicenistatin (**32**). In some events, sugar affected their bioactivity and pharmacological application. For instance, vicenistatin D-vicenisamine replaced with D-mycarose completely lost activity [107]. In the process of sugar biosynthesis, the substrate recognition of glycosyltransferase played a key role in the diversity of the products. Therefore, the modification of glycol groups in natural products often involved genetic manipulation of glycosyltransferases.

Auroramycin (**37**) containing a unique disaccharide motif was essential for its antimicrobial bioactivity. According to the putative sugar biosynthesis pathway, *kas*O*p was used and associated with CRISPR-Cas9-mediated genome editing by glycosyltransferase to regulate 3, 5-epi-lemonose biosynthesis [129]. Ultimately, specific alterations to the glycosylation pattern were made, and five analogues were synthesized. A comparative examination of activity revealed that the unique disaccharide moiety, namely C-methylation of the outer sugar unit, was critical for auroramycin’s antifungal action.

In the structures of sipanmycin A and B (**38**, **39**), the mode of the core structure with two sugar moieties were the same as auroramycin (**37**). In 2018, Mónica G. Malmierca et al. conducted an important experiment on the sipanmycin A and B’s route of biosynthesis and revealed that the cooperation of two different glycosyltransferases achieved the incorporation of the same sugar [23].

In addition, from Table 1, such kinds of compounds containing sugar groups were mainly explored using natural screening, and there was a scarce screening method targeting the glycosylation group. While this highlights the lack of a predictive model for using glycosylation chemistry for natural product optimization, it also suggested that effective glycol-diversification strategies, for example, chemo-enzymatic or bottom-up synthetic biology, can provide unique opportunities for improving and/or broadening natural product therapeutic potential [130].

## 5. Mechanism of Action

Polyene macrolactams (PMLs) are a unique class of substances in microbial metabolism. Many of them have biological activities such as antibacterial, cytotoxicity, regulation of Bcl-XL function [12] and the inhibition of adhesion of leukemia cells. Their specific mode of action in fungi had attracted the interest of researchers. The different binding sites can prevent cross-resistance between two drug classes with the same target [131].

Stubomycin (hitachimycin) (**49**) inhibited the synthesis of DNA, RNA and protein in yeast Saccharomyces cells in addition to causing holes on the surface of the cell membrane [18]. For specific performance inhibiting essential proton pump Pma1p, a P-type ATPase is a potentially effective therapeutic approach for fungal infection [18].

Leinamycin (LNM) (**48**) belongs to the family of polyene macrolactams and had a wide range of antimicrobial and antitumor activities. LNM, in particular, was demonstrated to have a distinct DNA-damaging mechanism of action involving activation by cellular thiols [132]. In the presence of sulfhydryl, a reductive cofactor, LNM, caused DNA alkylation, resulting in single-stranded DNA breaks [133]. Individual disulfide alkyl groups had different DNA shearing mechanisms in vitro compared with intact LNM [45].

Auroramycin (**37**) was a rare member of polyene macrolactams with antifungal bioactivity. The action of auroramycin in yeast cells was that it disrupted the structural organization of the vacuolar and the integrity of membranes, which differed from amphotericin B. Auroramycin was able to cause ionic stress and hyperpolarization of the yeast plasma membrane, which resulted in the microcosmic phenomenon of cation leakage from the cytoplasm [134].

Heronamide C (**25**) may have potential antifungal activities due to its saturated hydrocarbon chain and its ability to be inserted into the phospholipid bilayer: that is to say, passing the corresponding membrane phospholipid target. In the study, the authors detected the morphological changes of yeast cells after the compound treatment and speculated that heronamide C might induce the abnormal accumulation of cell wall substances by regulating the lipid function of the cell membrane and activating the membrane protein, and this eventually results in the changes in cell morphology [135,136].

The mechanisms of HASF (**83**) and analogues were different from other antifungal agents: that is, HASF interfering with sphingolipid biosynthesis in *Candida albicans* [137]. The mode of action of alteramide B was β-tubulin binding, which induced a ROS-mediated apoptosis pathway [70].

## 6. The Challenges and Opportunities of Current Research on PMLs

Antifungal medications included polyene macrolides such as amphotericin B and nystatin, as well as non-polyene-lactam lactamides, which were represented by penicillin. PMLs produced by binding polyene to the structural groups of macrocyclic lactams had a wide range of activities, and their various structural types may have unique active mechanisms, making them a kind of reserve prodrug for future human resistance to unknown diseases. However, for the time being, this family of chemicals was still in the early stages of the study, with some technical challenges that we would have to work through jointly.

### 6.1. Bottleneck Limitation for PMLs Isolation and Storage

The isolation and purification of polyene, as well as the identification of its absolute configuration, are problematic due to the instability of natural polyene products. Polyene macrocyclic lactamides possessing glycoside groups or carboxyl groups can exploit the hydrophilicity of the material itself by continually spreading the target from the aqueous phase to the organic phase following salt generation in the isolation and purification process. The targets were swiftly separated by a repeated partition of aqueous and organic phases, and other compounds with medium and long branched aliphatic chains showed obvious lipophilic characteristics. After concentration, precipitation and recrystallization of the crude extracts, a significant number of pure target products of the above listed were obtained [138].

Sceliphrolactam (**17**), micromololactam (**16**), FW05328-1 (**10**) and other PMLs contain monocyclic polyene lactam structures. There were many hydroxyl and methyl groups in the rings, but there were no additional branched chains similar to fatty chains or carboxyl groups. To obtain such compounds, microorganic extracts experienced numerous and complicated isolation steps. Because the compounds were easily degraded in the stimulation of light, temperature, heat and oxygen, the acquisition of large-scale highly purified samples would become critical in future research and application.

Fortunately, Christopher S et al. used sporopolleninexine capsules (SpECs) derived from the spores of the plant *Lycopodium clavatum* to extend the half-life of the compound and successfully isolated an active polyene antibiotic “marinomycin A” with a particularly short half-life in natural light in 2019 [138]. The SpECs had a porous shell structure with multidirectional, nanoscale channels that may encapsulate and release compounds in a regulated manner, and they can be employed as an appropriate medium for the storage and transport of photo-unstable polyene compounds. The findings provided new information on how to isolate and purify photo-unstable polyene molecules. Based on the structure of SpECs and other similar polymers, the core of the idea was to cross-link the corresponding groups with an affinity for polyene compounds in the interior of the porous shell structure to form artificial semi-synthetic specific materials similar to current macroporous resin materials, while also providing light protection and rapid and targeted separation of polyene compounds. This would have paved the way for large-scale procurement of polyene macrolactam compounds in the future, as well as their pharmaceutical application and medication delivery.

With further investigation, it was found that many natural small molecules or macromolecular proteins in the organic body were in a metastable/transition state. The molecule stayed in an inactive state as a complex with a protective agent (such as an amino group/protein); otherwise, the corresponding enzymes will remove the protective agent to activate the molecular. The extraction and purification of meta-stable molecular pose a challenge but we believed that with the acceleration of development and understanding of research on bio-macromolecule complexes, scientists will persistently approach and restore the truest condition and function of polyene macrolactams existing in vivo. In addition to the above-mentioned protective effect of artificial porous shell materials on polyene macrocyclic lactams, it is possible to combine protein substances and polyene macrocyclic lactam to form a stable complex for isolation, purification, preservation and application in the future.

### 6.2. PMLs Poor Solubility Blocking Bioactivities Assessment

Because the presence of hydrophobic groups such as medium and long aliphatic chains or amino acid phenylalanine in PMLs leading to their poor solubility, these affect BE-14106 (**22**), hitachimycin (**49**) and aureoverticillactam (**29**). These types of PMLs were difficult to use directly for the assessment of activities (katsuhisa kojiri *et al*., 1992; stubomycin., 1981). The problem of polyene macrolactam solubility could be solved by chemical semi-synthetic approaches in which the -OH group was attached to the sugar group or PEG, etc., to increase the solubility of compounds in the future.

### 6.3. Multidisciplinary Effort toward Further PMLs Discovery

#### 6.3.1. Innovation of Strain Source

The new source implied that there was a high likelihood of discovering chemicals with novel structures. Nevertheless, the possibilities of identifying new strains that can be cultured as mono-clones were dwindling within the confines of existing technologies. As a result, several researchers have explored the sky, seas and mines to find unusual natural strains and create novel methods for obtaining hitherto “difficult or un-cultivable” microbial strains. As a result, streaming single-cell separation and fluorescent marker combination of a single cell in situ culture techniques have been required for isolating the unusual, new species of microbial strains.

#### 6.3.2. Establishment of Chemical Tool Enzymology Library

Afterward, data collection about the key enzyme involved in each node of the novel PLM biosynthetic pathways was performed. Following that, heterologous expression, purification and the construction of the appropriate PKS enzymology library to produce several related high purity proteases from the PKS and NRPS biosynthetic routes were performed. Under mild in vitro conditions, the integration of enzyme engineering and chemical engineering was performed to execute or replicate biological substances synthesis processes in the body, i.e., constructing bionic biosynthesis.

From genes to proteins, the artificial transformation of the PKS enzyme system afforded the directionally known enzyme AT; the substrate specificity of adenosine, polyketone peptidase KQ, acyltransferase, transaminase, methyltransferase, methoxy-transferase, decarboxylase, thioester enzyme, glycosylation enzyme and glycosyl-transferase in the PKS natural products assembly process; and improved enzymology tools.

Specific matching and protein combinations would need to be made to ensure the smooth coordination function of the key nodes’ proteins in the PKS assembly line. To obtain the crystal structure [111,123] of biosynthetic enzymes X-ray diffraction, structural biology and physics methods were required. The surface interaction among proteins has already been observed using a Frozen Electron Microscope (FEM). A kinetics model for observing the movement of proteins should be built based on NMR data. A “high-speed cell machine” would be created to better understand the real kinetics mode of enzymes involved in the biosynthetic pathway and the relationship between enzymes and specific substrates. It will be more efficient in vitro and can lower the reaction barrier for the elucidation of new structures of synthetic compounds.

Currently, physics-based tools, such as X-ray crystal diffraction technology, have limited the establishment of biologic synthetase databases to some extent because the high purity, solubility and uniformity of protein crystal are difficult to obtain. It is necessary to design linkers artificially and add crosslinking and protective agents to stabilize the protein structure that meets detection demands, particularly for the protein complex in the transition state. These artificial components cannot be fully reproduced in the organism’s reaction process. Moreover, the crystal structure simply depicts the motion of the protein in a specific space and time frame. NMR technology can study the structure and motion state of protein molecules indestructibly, but it is limited to detecting proteins with a molecular weight of 20 KDa. The development of frozen electron microscopy (cryo-EM) technology has greatly broadened the scope of protein structure research, which will be no longer limited by the study of protein crystal structure but will be understood with the interface of protein interaction.

#### 6.3.3. Pharmacology and Mechanism of Bioactivity

Many PMLs draw much attention due to the multiple types of physiological activities and immunomodulatory activities. However, the mechanism for the bioactivity of PMLs remains insufficiently understood. Research on PMLs is also dependent on the progress and development of physics observation methods. With the advancement of bioinformatics and chemical informatics, biological fluorescein, isotope labelling technology, marking biological macromolecular protein via fluorescence microscope, microscope principle and scanning electron microscopy (SEM), drugs and proteins exist in the signal pathways of living organisms and can be visually and dynamically observed. This accelerates the understanding of the new compound structure and the mode of attachment of small molecules to a macromolecular protein, promoting the progress of mechanism research and providing assistance in new drug research and development processes. It is important to study the development of antifungal PMLs lead-drugs, which are currently in short supply in clinical trials.

## 7. Conclusions

This paper reviewed the discoveries, structural characteristics, producing strains, mechanism of actions, biosynthetic pathways and problems in the research of PMLs, as well as some possible solutions and research orientation. Hopefully, this paper will help scientists in exploring PMLs in the future.

In summary, due to the existence of active double-bond groups in PMLs, PMLs have fused into multiple groups to form a variety of structural classes and correspond to a variety of biological activities. Although the isolation and structural elucidation of these compounds have a long history, their biosynthesis pathways, especially those forming intramolecular cyclization, are still not clear. The study on in vivo peri-cyclic reactions and intramolecular cycloaddition chemical reaction mechanisms can better guide their chemical synthesis modification, as well as bionic synthesis.

## Data Availability

The data that support the findings of this review are openly available in references listed in this manuscript.
